# Complement Mediated Endothelial Damage in Thrombotic Microangiopathies

**DOI:** 10.3389/fmed.2022.811504

**Published:** 2022-04-25

**Authors:** Miquel Blasco, Elena Guillén-Olmos, Maribel Diaz-Ricart, Marta Palomo

**Affiliations:** ^1^Department of Nephrology and Kidney Transplantation, Hospital Clínic, Centro de Referencia en Enfermedad Glomerular Compleja del Sistema Nacional de Salud (CSUR), University of Barcelona, Barcelona, Spain; ^2^Institute of Biomedical Research August Pi i Sunyer (IDIPABS), Malalties Nefro-Urològiques i Trasplantament Renal, Barcelona, Spain; ^3^Hematopathology Unit, Department of Pathology, Hospital Clínic of Barcelona, Biomedical Diagnosis Centre (CDB), Institute of Biomedical Research August Pi i Sunyer (IDIBAPS), University of Barcelona, Barcelona, Spain; ^4^Barcelona Endothelium Team, Barcelona, Spain; ^5^Josep Carreras Leukaemia Research Institute, Hospital Clinic, University of Barcelona, Barcelona, Spain

**Keywords:** C5b-9 deposition, complement system activation, complement blockade, endothelia cells, membrane attack complex, thrombotic microangiopathies

## Abstract

Thrombotic microangiopathies (TMA) constitute a group of different disorders that have a common underlying mechanism: the endothelial damage. These disorders may exhibit different mechanisms of endothelial injury depending on the pathological trigger. However, over the last decades, the potential role of the complement system (CS) has gained prominence in their pathogenesis. This is partly due to the great efficacy of complement-inhibitors in atypical hemolytic syndrome (aHUS), a TMA form where the primary defect is an alternative complement pathway dysregulation over endothelial cells (genetic and/or adquired). Complement involvement has also been demonstrated in other forms of TMA, such as thrombotic thrombocytopenic purpura (TTP) and in Shiga toxin-producing *Escherichia coli* hemolytic uremic syndrome (STEC-HUS), as well as in secondary TMAs, in which complement activation occurs in the context of other diseases. However, at present, there is scarce evidence about the efficacy of complement-targeted therapies in these entities. The relationship between complement dysregulation and endothelial damage as the main causes of TMA will be reviewed here. Moreover, the different clinical trials evaluating the use of complement-inhibitors for the treatment of patients suffering from different TMA-associated disorders are summarized, as a clear example of the entry into a new era of personalized medicine in its management.

## Introduction

Thrombotic microangiopathies (TMA) constitute a group of disorders characterized by micoangiopathic hemolysis, platelet consumption and systemic organ damage. The identification of the underlying etiology among TMA-associated disorders is a major challenge due to the variability of clinical presentation and the absence of pathognomonic histological findings. Despite these factors, its assessment is crucial for directed therapy (1).

**FIGURE 1:**
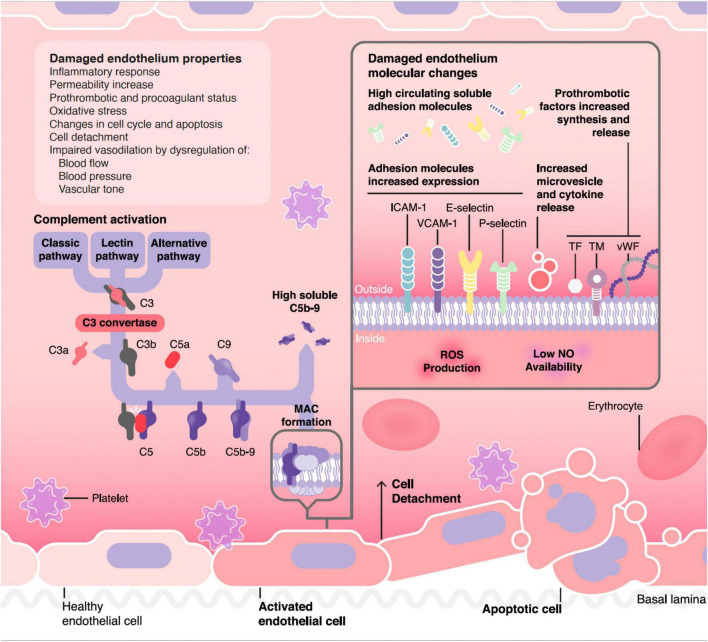
Pathophysiology of complement mediated endothelial damage in TMA. ICAM-1, Intercellular Adhesion Molecule 1; VCAM-1, Vascular cell adhesion protein 1; TF, tissue factor; TM, thrombomodulin; vWF, von Willebrand factor; MAC, membrane attack complex.

The vascular endothelium plays a pivotal role in the regulation of the hemostatic balance. In this regard, endothelial cells (EC) provide a non-thrombogenic inner layer of the vascular wall which maintains the blood fluidity, prevent thrombosis through different anticoagulant, and antiplatelet mechanisms, and regulate clot formation, limiting it to those areas without vascular integrity (2). Endothelial injury is the common underlying mechanism among different TMA, leading to the microvasculature thrombosis observed in these conditions (3) (Figure 1).

The complement system (CS) is a central component of innate immunity and bridges the innate to the adaptive immune response. Activation of complement pathways under physiological conditions facilitates the clearance of microbes, damaged cells and immune complexes from an organism, promotes inflammation, and attacks pathogen’s cell membrane. To prevent undesirable activation and tissue damage, complement activation is strictly controlled by numerous regulators under normal circumstances (4).

During the last decades, the contribution of dysregulated complement activation to endothelial damage has been widely demonstrated in some TMA forms. Among them, the prototype is atypical hemolytic syndrome (aHUS), in which complement dysregulation occurs as the primary event (genetic and/or adquired alternative complement pathway dysregulation). Complement involvement has also been demonstrated in thrombotic thrombocytopenic purpura (TTP) and in Shiga toxin-producing *Escherichia coli* hemolytic uremic syndrome (STEC-HUS), but, in these cases, it occurs as a secondary event, triggered either by ADAMTS13-deficiency or toxin-mediated injury, respectively (5). Finally, the role of complement has also been suggested to be involved in other TMA-associated disorders, although it has not been completely elucidated (6).

Distinguishing among the different causes of TMA may be challenging, leading to a delay in etiologic diagnosis and, therefore, in the initiation of the most appropriate treatment. The development of diagnostic tools based on functional and genetic studies to assess the involvement of complement in the pathogenesis of the different clinical entities is crucial (1). In this regard, new management approaches of TMA are being evaluated, and complement-targeted therapies are gaining importance since they selectively block this inflammation pathway, thus avoiding the side effects of some traditional therapies (7).

This review addresses the relationship between endothelial damage and complement dysregulation among the main causes of TMA. We also summarize the clinical trials carried out in TMA-associated disorders treated with complement inhibitors (Table 1) and the need to consolidate and develop different biomarkers that may allow an individualized treatment in the near future.

**TABLE 1:** Complement inhibitors clinical trials.

Drug	Function	Status	Phase	Description	Population	No. of participants	Identifier
**SHUa**							
Crovalimab	C5 inhibitor	Recruiting	3	Multicenter, single-arm study	Adults and adolescents	90	NCT04861259
Crovalimab	C5 inhibitor	Not yet recruiting	3	Multicenter, single-arm study	Pediatric patients	35	NCT04958265
Eculizumab	C5 inhibitor	Completed (May 30, 2017)	2	Open-label, Multi-center clinical trial	Adult, older adult	44	NCT01194973
Eculizumab	C5 inhibitor	Completed (April 29, 2015)	2	Open-label, multi-center clinical trial	Pediatric	22	NCT01193348
Eculizumab	C5 inhibitor	Completed (July 23, 2015)	2	Open-label, multi-center controlled clinical trial in patients with plasma therapy-resistance	Adults	16	NCT00844545
Iptacopan	Factor B inhibitor	Recruiting	3	Multicenter, single-arm, open label trial	Adult, older adult	50	NCT02604420
Ravulizumab	C5 inhibitor	Active, not recruiting	3	Single-arm study	Adults and adolescents	58	NCT02949128
Ravulizumab	C5 inhibitor	Active, not recruiting	3	Open-label, multicenter study	Children and adolescents	31	NCT03131219
**MAT related with autoimmune disorders**							
Ravulizumab	C5 inhibitor	Recruiting	2	Double-blind, randomized, placebo-controlled study in participants with proliferative lupus nephritis (LN) or immunoglobulin A nephropathy (IgAN)	Adults	120	NCT04564339
**Shiga-toxin Related SHUa**							
Eculizumab	C5 inhibitor	Completed (July 12, 2019)	3	Prospective randomized controlled therapeutic trial versus placebo	Pediatric	100	NCT02205541
Eculizumab	C5 inhibitor	Completed (April 5, 2013)	3	Open-label, multi-center trial	Child and adult	198	NCT01410916
**TMA after hematopoietic stem cell transplantation**							
Eculizumab	C5 inhibitor	Recruiting	2	Early intervention to treat TMA/aHUS-associated MODS	Children and young adults	21	NCT03518203
LFG316	C5 inhibitor	Terminated (January 5, 2021)	2	Randomized, open label, controlled, multiple dose study	Adult, older adult	7	NCT02763644
Ravulizumab	C5 inhibitor	Recruiting	3	Randomized, double-blind, placebo-controlled, multicenter study	Adults and adolescents	184	NCT04543591
Ravulizumab	C5 inhibitor	Recruiting	3	Open-label, single arm, multicenter study	Pediatric	40	NCT04557735
**Infections: COVID**							
AMY-101	C3 inhibitor	Not yet recruiting	2	Randomized, parallel assignment study	Adult, older adult	144	NCT04395456
Bradykinin	C1 inhibitor	Completed (August 18, 2021)	2	Prospective, randomized, double-blind, multicenter, prospective study	Adult, older adult	44	NCT05010876
Conestat Alfa	rhC1-INH	Active, not recruiting	2	Randomized, parallel-group, open-label, multi-center pilot trial	Adult, older adult	129	NCT04414631
Icatibant	C1 inhibitor	Recruiting	2	Randomized, open, multicenter, proof of concept	Adult, older adult	120	NCT04978051
Eculizumab	C5 inhibitor	Recruiting	2	Bayesian open labeled randomized clinical trial (nested in the CORIMUNO-19 cohort)	Adult, older adult	120	NCT04346797
Ravulizumab	C5 inhibitor	Recruiting	3	Open labe, randomized study	Adult, older adult	32	NCT04570397
Ravulizumab	C5 inhibitor	Terminated	3	Open-label, randomized, controlled study	Adult, older adult	202	NCT04369469
Ruconest	rhC1-INH	Recruiting	2	Randomized, parallel-group, open-label, multi-center pilot trial	Adult, older adult	120	NCT04530136
Zilucoplan	C5 inhibitor	Completed (July 2, 2021)	2	Prospective, randomized, open-label study	Adult, older adult	81	NCT04382755
**Infections: Sepsis**							
CaCP29	C5 inhibitor	Completed (April 25, 2016)	2	Randomized, placebo-controlled, double-blind, dose controlled trial	Adult, older adult	72	NCT02246595
C1-esterase inhibitor	C1-esterase inhibitor	Completed (December 2, 2014)	3	A randomized controlled pilot study	Adults	20	NCT01766414
**TMA associated with a trigger**							
Ravulizumab	C5 inhibitor	Recruiting	3	Randomized, double-blind, placebo-controlled, multicenter study in participants who have thrombotic microangiopathy associated with a trigger	Adult, older adult	100	NCT04743804

*For the resulting table several searches in clinicaltrial.gov were perform including all countries and interventional clinical studies that were completed, terminated, recruiting and not recruiting. Studies with less than 5 participants have been excluded. In those that are already completed the last update is included between parenthesis. The searches performed were: (1) combination of the concepts “thrombotic microangiopathy” and “complement inhibitors”, (2) the pathologies included in the present review and “complement inhibitors”, and (3) the search of several individual drugs.*

## Complement Endothelial Damage in Thrombotic Microangiopathies

### Atypical Hemolytic Uremic Syndrome

Dysregulation of the alternative complement pathway over EC surfaces is the main cause of TMA development in atypical hemolytic uremic syndrome (aHUS) (8, 9). Genetic variants in complement components (gain or loss function mutations) and/or acquired conditions (antibodies against complement factor H) have been identified in about 40–60% of patients (10–12). Coexistence of these predisposing factors with environmental triggers or complement amplifying conditions (i.e., infections, drugs, surgery, pregnancy) may lead to disease onset (13, 14). aHUS is an ultra-rare disease, that affects both pediatric and adult populations, of a systemic nature and with highly morbidity and mortality rates (14–16). These characteristics, together with the need for a clinical diagnosis (exclusion of all potential TMA causes), could delay an early etiological treatment with terminal complement blockers (17–22), decreasing organ function recovery outcomes (especially kidney function).

Endothelial activation and injury in aHUS occur mainly due to immune innate system dysregulation and the loss of protection at cell surfaces. Disease phenotype and severity will depend on pro- and anti-inflammatory cytokines balance and regulation of individual complement components (23, 24). Once the complement cascade is amplified, the terminal phase will play a key role, especially, the cleavage of C5 molecule into C5a and the subsequent generation of membrane attack complex (MAC), also called C5b-9 (16). C5a is a potent anaphylatoxin, promoting the recruitment of platelets and leukocytes to the endothelial surface. In addition, it causes endothelial retraction with the consequent exposure of the underlying basement membrane and overexpression of procoagulant tissue factor (25). MAC forms pores in pathogens or targeted cell membranes leading to osmolysis (cytolytic effector of innate and adaptative immunity). It can also induce cell activation and intracellular signaling (26), promoting a switch from an anti-coagulant and anti-inflammatory endothelial phenotype to a highly active, pro-coagulant phenotype. Although it may not be identified in all cases, aHUS patients present a defect of AP regulation over EC surfaces, allowing an abnormal formation of terminal complement phase (C5a and C5b-9) on the endothelium, which lead to cell apoptosis and TMA development, with a special predilection for the kidney vasculature (27).

### Thrombotic Thrombocytopenic Purpura

ADAMTS-13 (a disintegrin and metalloproteinase with a thrombospondin type 1 motif, member 13) deficiency is the key event in the pathophysiology of TTP (28). When functional, ADAMTS-13 cleaves the ultra-Large von Willebrand factor (ULVWF) multimers, which are highly reactive to platelets, before VWF is released to plasma. ADAMTS-13 deficiency, which can be congenital or acquired, leads to the accumulation of ULVWF secreted by the endothelium, causing platelet aggregation, formation of microthrombi and ultimately endothelial damage (29). However, an important factor in the pathogenesis of this disease is the different susceptibility of the vascular beds. The selective injury to dermal, renal, and cerebral microvasculature does not occur in EC from lung and liver (30). Notably, this different susceptibility is not appreciated in cells with a macrovascular origin. This phenomenon has not been described only *in vitro* experiments (31) but also in animal models (32), and offers the potential explanation for tissue distribution damage in TTP.

Although the role of complement dysregulation in TTP is not as well-defined as it is in aHUS etiology, it has been demonstrated through several approaches. Increased levels of complement anaphylatoxins and soluble C5b-9 (sC5b-9), have been detected in patients with acute TTP when compared with remission and also with healthy controls (33, 34). Furthermore, significant decrease of C3a and sC5b9 has been observed during plasma exchange in TTP patients (34). Higher complement activity has been also reported in patients dying during an acute TTP episode compared to patients responding to treatment (35). From the three different pathways through which the CS can be activated, the classical is the one triggered by immunocomplexes. The majority of TTP cases are idiopathic and mediated by immunoglobulin G antibodies to ADAMTS-13 (29). Therefore, antigen-antibody complexes formed by ADAMTS-13 and anti-ADAMTS-13 IgG directly activate the classical complement pathway, leading to downstream activation of C3 and formation of C3a (34). Moreover, the increased thrombi formation in acute TTP could also accelerate complement activation (36) through its activity as C5 convertase by forming positive feedback loops (37). In keeping with this, it has been documented that TTP could trigger aHUS in susceptible individuals presenting mutations in CFH or other complement-related proteins (38). Finally, there is evidence showing that anti-C5 therapy is effective in refractory TTP unresponsive to plasma (39).

In addition, the role of the lectin complement pathway (LP) has recently been postuated in TTP through the finding of mannose-binding lectin associated serine protease (MASP2) elevated levels in the sera from acute phase TTP patients. Moreover, *in vitro* experiments using plasma from PTT patients suggest a role of this pathway in microvascular endothelial cell injury through an specific caspase 8 activation that can be blocked by the anti-MASP2 human monoclonal antibody narsoplimab (40).

### Shiga Toxin-Producing *Escherichia coli* Hemolytic Uremic Syndrome

Shiga toxin (Stx)-producing *Escherichia coli* (STEC) O157:H7 is the main cause of hemorrhagic colitis, occurring mainly in childhood. In 5–15% of cases it progresses to hemolytic uremic syndrome (HUS), and constitutes the leading cause of HUS worldwide (41).

Endothelial damage plays a central role in the underlying pathogenesis of STEC-HUS, and Stx (principally Stx subtype 2) is thought to be the key in this microangiopathic process through several mechanisms. *In vitro* studies have demonstrated that Stx upregulates the generation of adhesive molecules (E-selectin, ICAM-1, and VCAM-1) and chemokines (MCP-1, IL-8, fractalkine), promoting the adhesion of leukocytes to cultured human EC under flow conditions (42, 43). Stx also induces rapid release of ULVWF multimers from EC and inhibits the multimer cleavage by ADAMTS13, thus favoring platelet adhesion and clot formation in the microvasculature (41, 44). Moreover, Stx promote changes in gene expression, stimulating mRNA and protein production of chemokines and cytokines which may exacerbate endothelial damage (45). All these events generate changes in the endothelial phenotype, resulting in a prothrombogenic intravascular environment.

Another key element in STEC-HUS pathogenesis is dysregulation of the CS, and, likewise, it is mainly driven by Stx. Stx activates complement in the fluid phase, with reduced plasma levels of C3 and augmented levels of C3a, Bb and sC5b-9 during the active phase of the disease (46, 47). This evidence, along with the demonstration in murine models of disease that factor B knockout mices were protected from Stx induced renal damage (48), indicates that complement activation is produced via alternative pathway (AP) in STEC-HUS patients. Moreover, deposits of C3 and C9 have been detected on platelet-leukocyte heterotypic aggregates and microvesicles obtained from these patients (49, 50), thus indicating that complement AP might contribute to endothelial damage and thrombosis in STEC-HUS.

Two double-blinded placebo controlled trials with eculizumab (C5 inhibitor) has been performed to evaluate its efficacy for STEC-HUS treatment: ECULISHU (NCT02205541) in France looking at renal outcome in pediatric population, completed in 2019, and ECUSTEC (NCT01410916) in the United Kingdom looking at overall disease severity, completed in 2015 (Table 1). Until results become available from these prospective, randomized control trials, practitioners will continue to rely on small case series, single-center studies, and case reports to guide the eculizumab off-label indication for STEC-HUS treatment.

### Transplantation Associated Thrombotic Microangiopathy

Transplantation associated thrombotic microangiopathy (TA-TMA) is a severe complication associated with allogeneic hematopoietic cell transplantation (HCT) that occurs when endothelial dysfunction triggers the CS inducing the formation of platelet enriched thrombi in the microvasculature and a consequent hemolytic anemia (51). This complication can constitute a mild self-limiting disease or be associated with multiorgan disorder leading to death (52). The incidence and mortality of TA-TMA varies widely among studies and hospitals due to heterogeneous diagnostic criteria and under-recognition (53).

The endothelial damage in HCT is multifactorial and cumulative (54, 55) due to the effect of the conditioning regimen, calcineurin inhibitors, infections, graft vs. host disease and processes inherent to HCT such as the engraftment syndrome, among others (56–59). This endothelial damage leads to a release of proinflammatory cytokines and procoagulant factors together with nitric oxide depletion and an increase in the expression of adhesion molecules at cell surface. All these phenomena promote further endothelial injury and leads to platelet aggregation and the initiation and propagation of the complement cascade (60, 61). There is evidence showing that neutrophil extracellular traps (NETs) could constitute the mechanistical link between endothelial damage and complement activation (62). Several approaches have identified increased levels of C3b and sC5b-9 (63, 64), being the last included in TMA diagnostic algorithm proposed by Jodele et al. (63). In this algorithm the authors assessed that TA-TMA should be suspected in HCT recipients with an acute elevation of LDH, proteinuria >30 mg/dL, and hypertension more severe than expected with calcineurin or steroid therapy, and that clinical interventions should be considered for patients with both proteinuria >30 mg/dL and elevated sC5b-9. All these phenomena have been included in the “Three-Hit Hypothesis” postulated by Dvorak et al. (65). This hypothesis includes the different risk factors for the development of TA-TMA in three potential consecutive events that culminate in a cycle of activation of endothelial cells: Hit 1, an underlying predisposition to complement activation (genetical susceptibility due to complement gene variants) or pre-existing endothelial injury; Hit 2, the direct effect on endothelium of toxic conditioning regimen; and Hit 3, the additional insults triggered by medications, alloreactivity, infections, and/or antibodies that occur post-transplant. Furthermore, the anti-C5 monoclonal antibody eculizumab has proven to be an effective therapeutic strategy for TA-TMA, both in pediatric (66) and adult (67) patients, supporting the involvement of CS in the pathogenesis of TA-TMA.

### Kidney Transplantation Associated Thrombotic Microangiopathies

TMA associated with kidney transplantation may occur as a recurrence of aHUS or without previous history of thrombotic microangiopathy (*de novo* TMA). Kidney transplantation recipients are exposed to many triggers that can damage the endothelium and produce TMA, which can have a significant impact on allograft survival (68). The incidence is concentrated in the first months after transplantation and the main causes are ischemia-reperfusion damage, acute humoral rejection, medications, and opportunistic infections (69). Unlike TMA that occurs in native kidneys, graft biopsy is very useful since it can offer an etiological diagnosis in most cases.

aHUS recurrence after transplantation depends on genetic predisposition and endothelial damage is mediated by a dysregulation of AC pathway (70, 71). Endothelium could also be injured by important number of factors in transplanted patients, triggering *de novo* TMA. Calcineurin inhibitors (CNI) can induce TMA through arteriolar vasoconstriction (increased synthesis of endothelin and thromboxane A2, and reduced expression of prostacyclin and prostaglandin E2) (72–74), platelet activation, anti-fibrinolytic and pro-coagulant effects (75) and, by activation of AC pathway (microparticles released by injured EC) (76). m-TOR inhibitors block cell cycle progression and proliferation, induce endothelial progenitor cells death, increase procoagulant status, reduce fibrinolytic state and decrease renal expression of endothelial growth factor (77–80). All these processes can contribute to the endothelial damage that triggers *de novo* TMA onset, described with the use of CNI, m-TOR, or a combination of both (possibly related to elevated serum levels) (81, 82). Acute humoral rejection is an important cause of endothelial damage in kidney grafts, frequently associated with TMA development. Donor specific antibodies bind to major histocompatibility complexes on EC, fixing the CS and activating them through the over-expression of pro-inflammatory genes. Terminal complement phase (C5b-9) has been also related to allograft vasculopathy development in humoral response, since it can activate EC, favoring T cell recruitment and secretion of cytokines and interferon γ. Finally, ischemia-reperfusion injury is also associated with endothelial damage and TMA onset. The changes induced on EC produce complement activation that in turn accentuates and perpetuates endothelial damage at the expense of MAC (83). Animal models have shown a clear benefit of blocking the terminal phase of complement by different therapeutic strategies (84–86).

### Thrombotic Microangiopathies Related to Autoimmune Disorders

Antiphospholipid syndrome (APS) and systemic erythematous lupus (SLE) are the autoimmune disorders most frequently associated with TMA, which can result in a life-threating complication in these contexts.

APS is a prothrombotic disorder characterized by thrombosis and/or pregnancy morbidity, which occurs in the presence of antiphospholipid antibodies (aPL). Among them, anti-β2-glycoprotein I (B2GPI) antibodies are one of the main drivers of the endothelial damage, since they upregulate the endothelial expression of prothrombotic factors and adhesion molecules. B2GPI also interacts with von Willebrand factor (vWF), leading to platelet activation (87). Moreover, murine models have demonstrated that increased complement activation plays a role in APS pathogenesis, and the interaction of C5a with its receptor C5aR leads to inflammation, placental insufficiency, and thrombosis (88, 89). APS can occur in isolation or in association with other autoimmune diseases, such as SLE.

SLE is a multisystem autoimmune disorder associated with the presence of autoantibodies against double-stranded DNA (ds-DNA), among others, which can produce endothelial damage, either directly binding to EC or forming circulating immunocomplexes that deposit on vessels. In this regard, it has been demonstrated that several indicators of endothelial dysfunction (Pentraxin 3, E-selectin, VCAM-1) are higher in patients with SLE compared to healthy controls (90). CS is also involved in SLE pathogenesis, since these immunocomplexes activate the classical complement pathway and cause tissue damage, as can be observed in kidney biopsies of patients with lupus nephritis, in which C1q, C3 and C4d deposition is common (90, 91). Moreover, there is increasing evidence that CS is linked with thrombotic events occurring is SLE. An example is the relationship that has been observed between the deposition of complement activation products (C1q, C3d, C4d) on platelets surfaces with an increased risk for venous thrombosis in SLE patients (92).

### Thrombotic Microangiopathies Associated With Infections

Besides the above-mentioned STEC-HUS, TMA has been associated with a large number of infectious diseases, specially viral (6). Mechanisms of TMA-associated with infectious diseases are complex, and differ depending on the pathogens causing them.

Cytomegalovirus (CMV) can directly damage EC and cause platelet adhesion by inducing the expression of adhesion molecules and release of vWF (93). Moreover, CMV activates the complement classical pathway by binding of C1q to CMV infected cells (94).

TMA is also a known complication of HIV infection, with different forms of presentation. Cases of both classic TTP and aHUS have been described in HIV-infected patients (95, 96), although the exact mechanisms involved remain unclear. Most cases present without a severe reduction in ADAMTS-13 activity levels, which support the hypothesis that the underlying mechanism may be different from classic TTP. It has been suggested that endothelial cells can be infected by HIV (96).

Moreover, TMA induced by influenza A virus (H1N1) infection was reported during the 2009 pandemic. The neuraminidase produced by this virus, as well as by the bacteria *Streptococcus pneumoniae*, causes erythrocyte fusion and hemolysis, activation of platelets and generation of thrombin, leading to TMA in both cases. Furthermore, low levels of ADAMTS-13 and elevated sC5b-9 have been detected in patients with H1N1 infection (97).

More recently, the severe acute respiratory syndrome coronavirus 2 (SARS-COV-2) has emerged as another clear example of viral infection in which endothelial damage occurs in parallel to an overactivation of complement cascade. Patients with moderate and severe COVID-19 disease exhibit elevated C5a and sC5b-9 plasma levels compared with healthy controls, the later correlating with vWF plasma levels and disease severity (98). Moreover, COVID-19 patients were also found to have C5b-9 and C4d skin deposits, as well as mannose-binding lectin (MBL) deposits in lungs, suggesting the overactivation of alternative and lectin-complement pathways (99–101). These observations may reflect the close relationship between endothelial stress and complement dysregulation in this condition. In this regard, isolated experiences using complement inhibitors under a compassionate-use program, such as narsoplimab (102), or AMY-101, a compstatin-based C3 inhibitor (103) has been publicated. The former (narsoplimab) demonstrated rapid and sustained reduction of circulating endothelial cells, as well as with decreased circulating inflammatory cytokines, while the second (AMY-101) was associated with a favorable clinical evolution in a patient with COVID-19 severe pneumonia. All together, these results suggest that CS plays a central role in the pathophysiology of COVID-19-related lung injury.

Moreover, several clinical trials targeting the complement system are currently ongoing in patients with COVID-19 (Table 1). Among them, two have been completed: AntagoBrad-Cov (NCT05010876), a prospective, randomized, double-blind, multicenter study of three parallel groups of patients comparing the efficacy of human C1 inhibitor, administered alone or in combination with icatibant (a specific bradykinin B2 receptor antagonist) on the pulmonary manifestations of COVID-19 infections; and ZILU-COV (NCT04382755), a prospective, randomized, open-label, study to investigate the efficacy of a complement C5 inhibitor (Zilucoplan^®^) in improving oxygenation and short-and long-term outcome of COVID-19 patients with acute hypoxic respiratory failure. The results of the latter haven’t been published yet.

Interestingly, samples from septic shock (SS) patients induced higher C5b-9 deposition on EC than those from COVID-19 patients, whereas there were no differences regarding sC5b-9 levels between both groups. Thus, COVID-19 endotheliopathy may differs from SS, in which endothelial damage and complement may also play an important pathogenic role (98). In this regard, two clinical trials have been performed (Table 1). The first one, completed in 2014, is VECTORII Study (NCT01766414), a randomized, controlled, pilot study to evaluate the role of a C1-esterasa inhibitor in the modulation of innate immune response in a human endotoxemia model. The inhibition of C1-esterase exerted anti-inflammatory effects in the absence of classic complement activation (104). The second one, completed in 2016, is SCIENS Study (NCT02246595), a phase II clinical trial conducted to study safety, tolerability, pharmacokinetics, and pharmacodynamics of Vilobelimab (IFX-1; CaCP 29), a recombinant monoclonal antibody against C5a, in patients with severe sepsis or septic shock. Vilobelimab demonstrated to neutralize selectively C5a in a dose-dependent manner without blocking formation of the membrane attack complex, and without resulting in detected safety issues (105).

## Discussion

Clinical TMA management (diagnostic-therapeutic process) is a great challenge due to its systemic nature (variable signs and symptoms) and high associated morbidity and mortality. Enhancing the etiological knowledge of the different clinical entities involved in TMA will allow the development of targeted therapies that may improve their poor prognosis. The cornerstone for thrombi development in the microvasculature is the endothelial cell damage, common to all TMA-associated disorders. However, these pathological processes exhibit differential patterns and mechanisms of endothelial injury, although occasionally there may be also a pathophysiological overlap.

CS, a key element of the innate immune system, requires precise regulation. Defects in the elements involved result in dysregulation and over-activation. In the case of TMA, an acquired or congenital dysregulation of the alternative complement pathway is primarily responsible for the endothelial damage that occurs in aHUS patients. Therefore, the development of monoclonal antibodies that block the terminal complement phase (C5) has led to a great advance in aHUS management, especially with early therapy introduction. The excellent response to these treatments has led to the research of the potential pathological role of the CS in other TMA forms with higher prevalence and without known etiological treatment.

Figure 1 summarizes the pathophysiological process through which complement could damage the endothelium in different TMA forms, as well as the resulting functional and molecular endothelial changes. One of the difficulties faced in making a diagnosis of complement mediated TMA is the lack of reliable markers of CS hyperactivation. For instance, in the case of the paradigmatic complement mediated TMA, aHUS, relatively few patients have consumption of complement factors, complement mutations are heterozygous-and the corresponding protein concentrations in blood are not consistently abnormal- and genetic screenings are slow and even uninformative in up to 50% of cases (106). In general, the quantification of levels of individual complement components in serum is not a straightforward approach in TMA diagnosis, as soluble levels are not reliable biomarkers of complement activation in any form of TMA (107), although the quantification of sC5b-9 has shown some usefulness in the management of patients with TA-TMA (64) and is available clinically (commercial kits). For this reason, new diagnostic tools are needed for TMAs management. In this regard, functional studies may help to demonstrate the role of complement activation not only at diagnosis, but also to monitor treatment response. Among them, the analysis of C5b-9 deposition on endothelial cells culture could be an attractive option, since it has shown a great correlation in different clinical stages of aHUS patients, as well as in certain secondary TMA forms by different research groups (108–112). However, there is still an open discussion about their utility, especially because it is not a quick procedure, requires specialized and trained personnel, and is not commercially available (1).

In section 2, we have reviewed the evidence generated regarding the participation of the CS in various TMA forms. Since the approval of eculizumab for the treatment of aHUS in 2011, the development of therapies that directly target CS at different levels has enabled a large number of clinical trials focused on complement blockers in patients with different TMA-associated disorders (Table 1). Most of them have evaluated the role of C5-inhibitors, mainly eculizumab and ravulizumab (a long-acting C5-inhibitor), in the treatment of various conditions. Among them, those performed with eculizumab in patients with aHUS and STEC-HUS have been already completed. In recent decades, a large number of potential complement-related targets have progressively emerged, such as C3, factor B and C1q. Probably, this fact may be related with the large amount of scientific evidence generated regarding the role of complement in the pathogenesis of the different conditions reviewed in this work. As shown in Table 1, several clinical trials evaluating these factors are ongoing, and their results may probably help to extend the therapeutic options for TMA-associated conditions in the near future.

In conclusion, endothelial damage in TMA can have different origins depending on the responsible pathological process. However, the potential role of complement system in many of them represents a real and current treatment opportunity. Identifying the exact factors involved in each disease will allow an individualized patient management. This is a challenge that will require a great effort involving clinicians, immunologists, geneticists, and basic researchers.

## Author Contributions

MB reviewed the bibliography and wrote aHUS, kidney transplantation associated TMA and discussion sections. MP reviewed and wrote the TTP and TA-TMA sections, and designed Table 1 and Figure 1 with the support of MB and EG-O. EG-O reviewed and wrote introduction, STEC-HUS, TMA related to autoimmune disorders, and TMA associated with infections sections. MD-R contributed to the manuscript with her critical review and comments. All authors contributed to the article and approved the submitted version.

## Conflict of Interest

MB reports advisory boards and symposium speaker honoraria from Alexion Pharmaceuticals. MD-R has received research funding and speaker fees from Jazz Pharmaceuticals. The remaining authors declare that the research was conducted in the absence of any commercial or financial relationships that could be construed as a potential conflict of interest.

## Publisher’s Note

All claims expressed in this article are solely those of the authors and do not necessarily represent those of their affiliated organizations, or those of the publisher, the editors and the reviewers. Any product that may be evaluated in this article, or claim that may be made by its manufacturer, is not guaranteed or endorsed by the publisher.
